# Carbon-Based Composites with Mixed Phosphate-Pyrophosphates with Improved Electrochemical Performance at Elevated Temperature

**DOI:** 10.3390/ma16196546

**Published:** 2023-10-04

**Authors:** Sonya Harizanova, Trajche Tushev, Violeta Koleva, Radostina Stoyanova

**Affiliations:** Institute of General and Inorganic Chemistry, Bulgarian Academy of Sciences, 1113 Sofia, Bulgaria; sonya@svr.igic.bas.bg (S.H.); tushev@svr.igic.bas.bg (T.T.)

**Keywords:** sodium-ion batteries, lithium-ion batteries, hybrid metal-ion batteries, Na_4_Fe_3_(PO_4_)_2_P_2_O_7_, carbon composites, rGO, synthesis, structure, characterization

## Abstract

Sodium iron phosphate-pyrophosphate, Na_4_Fe_3_(PO_4_)_2_P_2_O_7_ (NFPP) emerges as an excellent cathode material for sodium-ion batteries. Because of lower electronic conductivity, its electrochemical performance depends drastically on the synthesis method. Herein, we provide a simple and unified method for synthesis of composites between NFPP and reduced graphene oxide (rGO) and standard carbon black, designed as electrode materials for both sodium- and lithium-ion batteries. The carbon additives affect only the morphology and textural properties of the composites. The performance of composites in sodium and lithium cells is evaluated at elevated temperatures. It is found that NFPP/rGO outperforms NFPP/C in both Na and Li storage due to its hybrid mechanism of energy storage. In sodium half-cells, NFPP/rGO delivers a reversible capacity of 95 mAh/g at 20 °C and 115 mAh/g at 40 °C with a cycling stability of 95% and 88% at a rate of C/2. In lithium half-cells, the capacity reaches a value of 120 mAh/g at 20 and 40 °C, but the cycling stability becomes worse, especially at 40 °C. The electrochemical performance is discussed on the basis of ex situ XRD and microscopic studies. The good Na storage performance of NFPP/rGO at an elevated temperature represents a first step towards its commercialization.

## 1. Introduction

Mixed phosphate-pyrophosphates of sodium and transition metals, Na_4_M_3_(PO_4_)_2_P_2_O_7_ (M = Fe, Mn, Co, Ni), emerge as excellent cathode materials for sodium-ion batteries due to their flexible structure and ability to adopt ions in different oxidation states [1,2,3]. Given the two building polyanion units, namely PO_4_^3−^ and P_2_O_7_^4−^ groups, the manner of their connectivity yields an unique structure matrix which ensures a three-dimensional insertion pathway with a low activation energy barrier for both smaller Li^+^ and bigger K^+^ via Na^+^ [2,3,4,5,6,7,8]. In addition, the interconnectivity of PO_4_^3−^ and P_2_O_7_^4−^ groups contributes to high structure stability and easy alkaline ion diffusion owing to a more open framework [5,9].

Among mixed phosphate-pyrophosphates, the iron analogue, Na_4_Fe_3_(PO_4_)_2_P_2_O_7_, operating at about 3 V vs. Na^+^/Na, is considered to be the most attractive material for large scale applications owing to its low cost, natural abundance and reasonable energy and power density [5,6,10,11,12,13]. After the first report of Kang and coworkers [2], numerous studies have been directed to improve the poor cycling and rate performance of Na_4_Fe_3_(PO_4_)_2_P_2_O_7_ (hereafter NFPP) mainly through particle design and coating with various carbon materials, including reduced graphene oxide (rGO), in order to increase the material conductivity and overcome the sluggish Na^+^ diffusion kinetics [6,10,11,14,15,16]. Carbon-coated NFPP obtained by a solution combustion synthesis shows a comparatively stable cycling at 1 C rate with a loss of capacity from 95 mAh/g to about 80 mAh/g after 200 cycles and 100% Coulombic efficiency [6]. The research group of Cao et al. has developed effective methods (such as a template approach using triblock copolymer F127 as surfactant and spray drying) for preparation of NFPP/C nanospheres and microspheres NFPP@rGO with a longer cycle life even at high current densities [10,14]. NFPP/C nanospheres (~30 nm) coated with carbon layer (~3 nm) deliver a high discharge capacity of 128.5 mAh/g (near to the theoretical capacity of 129 mAh/g) at C/5 and capacity retention of 63.5% at 10 C after 4000 cycles [10]. The excellent cycling stability is combined with an excellent rate capability, even at such ultrahigh current rates of 80 and 100 C, where the achieved capacities are 90.0 and 79 mAh/g, respectively. The addition of rGO into the initial solution during the spray-drying synthesis procedure results in the formation of 3D graphene-decorated Na_4_Fe_3_(PO_4_)_2_P_2_O_7_ microspheres (NFPP@rGO) with high reversible capacity of 128 mAh g^−1^ at C/10, a long cycling life over 6000 cycles at 10 °C with 62.3% capacity retention and superior rate capability of 35 mAh/g at 200 C [14]. A recent study of Chen et al. demonstrates the great potential of Na_4_Fe_3_(PO_4_)_2_P_2_O_7_ for commercial application towards SIB [16]. They have offered an economical method to prepare a rust-derived NFPP/C composite with a porous and hollow spherical morphology (HS-NFPP/C) which ensures a shortening in the Na^+^ diffusion pathway, facilitates fast electron transport as well as efficiently accommodates the volume changes upon charge/discharge reactions. This material exhibits outstanding cycling performance in both Na half-cell and Na full battery (pouch cell) which has never been reported before for iron-based polyanionic cathodes [16]. Thus, in Na half-cell, it displays a reversible capacity of 69.5 mAh/g at 10 C with a capacity retention of 87.5% after 10,000 cycles as the operating voltage retains above 2.98 V vs. Na/Na^+^. The full battery, using hard carbon as the anode, delivers a discharge capacity of 2761 mAh at 0.5 C after 1000 cycles, corresponding to a high-capacity retention of 93.3%. Moreover, HS-NFPP/C is able to fast Na-ion intercalation/deintercalation reactions which is evident by the achieved reversible capacities of 86.5, 68.9, and 57.1 mAh/g at rates of 1, 50, and 100 C, respectively [16].

A limited number of papers report on the operation of carbon composites of NFPP (with carbon black and soot) in hybrid Na/Li electrochemical cells [15,17,18]. When cycling in Li cells, a competitive co-intercalation of Li^+^ and Na^+^ occurs at the cathode side with formation of the mixed Na_4−x_Li_x_(PO_4_)_2_P_2_O_7_ compositions [15,17,18]. In these mixed compositions, a part of the Na^+^ ions (about 1–1.2 Na^+^ per formula unit) always remains in the NFPP structure (the final composition being Li_3_Na(PO_4_)_2_P_2_O_7_) and it is responsible for its stability [2,4,17]. Kosova et al. [17] have established that the initial capacity in the Na cell with NaClO_4_ electrolyte is higher than that in Li cell with LiPF_6_ electrolyte: 104 vs. 95 mAh g^−1^ at C/5 rate, but the rate capability and reversibility are better in the Li cell—on going from rate C/10 to 1 C, the decrease in the discharge capacity is 40% vs. 20% in the Na and Li cell, respectively. Furthermore, the increase in the Na concentration in the dual Na/Li electrolyte is found to improve significantly the electrochemical performance of NFPP/C, which is explained by some competitive processes with participation of Na^+^ and Li^+^ (different conductivities in LiPF_6_ and NaPF_6_, higher polarizing ability of Li^+^ than Na^+^, and higher diffusion coefficient of Li^+^ in the solid state than that of Na^+^) [18]. The effect of Li electrolyte on the electrochemical behavior of NFPP/C has also been examined and it is shown that the Li^+^ intercalation proceeds to a higher extent in the LiTFSI-based electrolyte than in LiPF_6_ [15]. In sodium cells with an imide-based electrolyte, the cycling stability and rate capability outperform those in lithium ion cells with imide-based electrolytes [15].

The use of an NFPP@rGO cathode results in the improvement of the K^+^ storage properties in comparison with in situ carbon-coated NFPP/C [6,7]. An NFPP/C material prepared via the solution combustion method is able to intercalate reversibly K^+^ with a capacity of 116 mAh/g at C/20 rate and after 100 cycles at 1 C rate, the capacity is still about 70 mAh/g [6], while NFPP@rGO delivers an initial capacity of 80.3 mAh/g at 2 C current density and retains 82.1% of this capacity after 500 cycles [7].

The benefits of the rGO decoration have been established in other mixed polyanionic cathode materials such as Na_4_Ni_3_(PO_4_)_2_P_2_O_7_/rGO [19], Na_3_V_2_(PO_4_)_2_F_3_@C@rGO [20] and NaFe_2_(PO_4_)(SO_4_)_2_ @rGO [21]. Two groups, Xia et al. [22] and later Cao et al. [23], have focused on another iron-based mixed phosphate–diphosphate with close chemical composition to NFPP, namely Na_3_Fe_2_(PO_4_)P_2_O_7_ and reported results for excellent cycling stability and rate capability of Na_3_Fe_2_(PO_4_)P_2_O_7_/rGO composites obtained via spray drying. At a 20 C rate, the material of Xia et al. [20] shows a capacity of 55 mAh/g after 6400 cycles (89.7% capacity retention), while that of Cao et al. [23]—51 mAh/g after 8000 cycles (72.4% capacity retention). In addition, Cao et al. have studied the electrochemical performances of Na_3_Fe_2_(PO_4_)P_2_O_7_/rGO and Na_3_Fe_2_(PO_4_)P_2_O_7_/C and the comparison shows that the rGO-based composite overperforms the C-based one in terms of achieved reversible capacity at different current densities (from 1 C to 100 C) and cycling stability [23]. Better electrochemical performance in the presence of rGO was explained by the enhanced sodium ion diffusion kinetics in Na_3_Fe_2_(PO_4_)P_2_O_7_/rGO than that of Na_3_Fe_2_(PO_4_)P_2_O_7_/C as revealed by the Na^+^ diffusion coefficients within the composite electrodes.

All these findings clearly manifest the effectiveness of the carbon decoration and particularly with rGO for improved cycling and rate performances of Na_4_Fe_3_(PO_4_)_2_P_2_O_7_ positive electrodes. For practical application, there is a need to elaborate a simple and reproducible method for coating of NFPP with carbon-based materials. In addition, the operation of NFPP-electrodes at elevated temperatures is also of importance for commercialization. However, these studies are still scarce. To the best of our knowledge, only two papers report on the effect of the operating temperature on the electrochemical performance of Na_4_Fe_3_(PO_4_)_2_P_2_O_7_/C composites [11,24]. According to the results of Yang et al. [24], slightly higher discharged capacities and better rate capability (from 0.2 C to 10 C rate) have been achieved at an elevated cell temperature of 55 °C: for instance, a discharge capacity of 78 mAh/g at 25 °C vs. 82 mAh/g at 55 °C at 10 C rate. W. Chen et al. have tested NFPP/C composites at three cell temperatures: −20 °C, 20 °C, and 50 °C (all-climate condition) [11]. Their data show that there is almost no difference between room temperature and 50 °C in respect to achieved capacities and rate performances. At low temperature (−20 °C), the capacity decreases in comparison with that at 50 °C (95 mAh/g vs. 110 mAh/g at C/10), as well as a fast capacity drop being established with increased current density.

Herein, we provide a simple and unified method for the synthesis of composites between NFPP and carbonaceous materials designed as electrode materials for both sodium- and lithium-ion batteries. The synthetic method consists in the ball-milling of NFPP with carbonaceous materials followed by thermal treatment at 400 °C in Ar atmosphere. Two types of carbonaceous materials are selected: rGO and standard carbon black additives. The performance of NFPP/rGO and NFPP/C in sodium and lithium half-cells is evaluated at elevated temperatures in the framework of CV and CDG experiments. Through detailed diffraction, spectroscopic, and microscopic tools, the changes in composites at pristine state and after electrochemical reactions are monitored.

## 2. Materials and Methods

### 2.1. Materials and Synthesis

High-purity powder of NaH_2_PO_4_·H_2_O (ACS, Sigma-Aldrich, Rockford, IL, USA) and Fe(HCOO)_2_·2H_2_O prepared by us were used as starting materials. The crystals of Fe(HCOO)_2_·2H_2_O were synthesized via dissolution of metal iron in 40% solution of formic acid (Merck, Darmstadt, Germany) at about 60–70 °C, followed by the solution concentration and filtration of the crystals [25]. As conductive additives, we utilized Super C65 carbon black (TIMCAL Ltd., Bodio, Switzerland) and reduced graphene oxide (rGO) provided by Graphit Kropfmühl GmbH (Hauzenberg, Germany).

Na_4_Fe_3_(PO_4_)_2_P_2_O_7_ was prepared after a thermal treatment at 500 °C of a freeze-dried phosphate-formate precursor containing NaH_2_PO_4_·H_2_O and Fe(HCOO)_2_ (4:3 mole ratio). The details for the synthesis can be found elsewhere [15].

Two types of carbon composites were prepared using simple, one-step ball-milling of NFPP with 15 wt.% carbon black and 15 wt.% rGO (labelled NFPP/C and NFPP/rGO, respectively). The ball-milling process was performed via means of planetary mono mill “Pulverisette 6” (Fritsch GmbH, Idar-Oberstein, Germany) with agate balls having ϕ of 10 mm, powder to balls mass ratio is 1:10, the duration being of 4 h at a speed of 300 rpm. Thus, obtained composites have been annealed at 400 °C for 3 h under Ar flow.

### 2.2. Methods

The structure of NFPP, carbon composites, and cycled cathodes was determined by powder X-ray diffraction (Bruker Advance D8 diffractometer, Karlsruhe, Germany) with LynxEye detector (CuKα radiation). The lattice parameters were calculated by WinPLOTR program. The local structure of anionic groups was analyzed by IR spectroscopy. The FT-IR spectra were recorded on a Nicolet Avatar-320 FTIR spectrometer (Thermo Fisher Scientific, Waltham, MA, USA) in KBr pellets (resolution < 2 cm^−1^). The specific surface area of the powder samples was measured using the BET method from low-temperature adsorption–desorption isotherms (77.4 K) recorded via NOVA 1200e device (Quantachrome, Boynton Beach, FL, USA). The total pore volume was calculated according to Gurwitsch’s rule at p/po = 0.99 and the pore size distribution was estimated according to the Barett–Joyner–Halenda (BJH) method. The pore size distribution for rGO was calculated using the DFT model. The morphology of powder samples and electrodes was studied via scanning electron microscopy (SEM) using JSM 6390 microscope (JEOL, Tokyo, Japan). TEM analysis was performed with a JEOL 2100 microscope (Tokyo, Japan) with a GATAN Orius 832 SC1000 camera (Plesantan, CA, USA). The optical images of the electrodes were obtained using ZEISS Stemi 508 stereo microscope (Carl Zeiss, Jena, Germany). 

The electrochemical characterization was carried out in sodium and lithium half-cells in galvanostatic and potentiostatic regimes by means of 32 channel Biologic VMP-3e battery cycler (Seyssinet-Pariset, France) at 20 and 40 °C (KB-53 incubator, Binder GmbH, Tuttlingen, Germany). The positive electrodes, supported using aluminum foil, were made from mixtures containing 80 wt.% composite, 10 wt.% Super C65 carbon and 10 wt.% polyvinylidene fluoride (PVDF) (Sigma-Aldrich, St. Louis, MO, USA). The slurry was then cast with a doctor blade film coater (ZAA 2600.A, Proceq SA, Schwerzenbach, Switzerland) onto the aluminum foil, followed by a drying at 80 °C overnight and the resulting electrode film is cut into 10 mm discs, pressed and dried at 120 °C under vacuum for 10 h. The electrode loading is around 8 mg/cm^2^ which corresponds to a thickness of about 300 μm. The negative electrodes consist of sodium and lithium metals. The electrolytes are 1 M NaPF_6_ in PC (Sigma Aldrich, St. Louis, MO, USA) and 1 M LiPF_6_ (EC/DMC, 1:1 by volume, Sigma Aldrich, St. Louis, MO, USA). Swagelok type sodium and lithium half-cells were assembled in an argon-filled glovebox (MB-Unilab Pro SP (1500/780), H_2_O, and O_2_ content < 0.1 ppm, MBraun, Garching, Germany) using Whatman GF/D (Whatman International Ltd., Maidstone, UK) as the separator. The galvanostatic cycling was performed over the range 1.5–4.5 V vs. Na^+^/Na and 1.5–4.8 V vs. Li^+^/Li at a rate of C/2 (1 C = 129 mA/g), while the cyclic voltammetry measurements were performed with a scanning rate of 1 mV/s in the range of 1.5–5 V vs. Na^+^/Na or Li^+^/Li. The specific capacity was calculated based on the mass of the active NFPP phase in the electrodes. For ex situ analyses, the electrodes were switched off at 1.5 V and the electrochemical cells were disassembled inside the glove-box; the electrodes were covered with plastic film and then subjected to different analyses.

## 3. Results and Discussion

### 3.1. Structure and Morphology Characterization of NFPP/C and NFPP/rGO Composites

**Figure 1:**
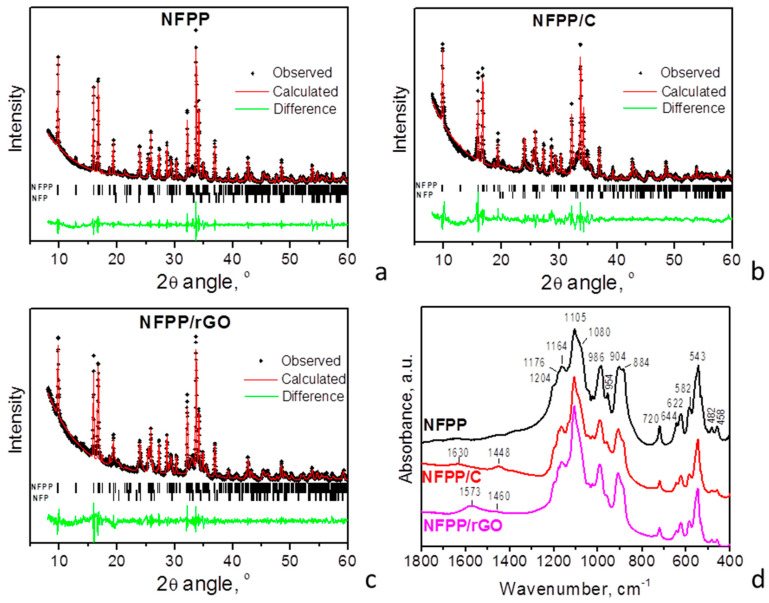
XRD patterns of pristine NFPP and composites NFPP/C and NFPP/rGO (**a**–**c**). “NFP” in the figures denotes the diffraction peaks due to maricite NaFePO_4_. FT-IR spectra of NFPP, NFPP/C, and NFPP/rGO (**d**).

Figure 1 compares the XRD patterns of pristine NFPP and its composites with carbon black and rGO. For all composites, the diffraction peaks are indexed in the space group *Pn*2_1_*a*, which is identical to that for pristine NFPP. Moreover, the unit-cell parameters also remain intact (Table 1). This indicates that NFPP in both composites NFPP/rGO and NFPP/C preserves the NASICON-type crystal structure of the pristine NFPP. The XRD peak profiles also appear to be unchanged after the formation of composites, thus evidencing the retention of the crystallinity of NFPP. In addition to the main NASICON phase, low-intensive peaks due to maricite phase, NaFePO_4_, are also distinguished irrespective of the type of carbon additives. This means that the impurity phase, already formed during the synthesis of pristine NFPP (including freeze-drying reactions followed by thermal treatment at 500 °C), is not affected after the ball milling with carbon additives. It is worth mentioning that, irrespective of the synthesis methods, NFPP formation is always accompanied with impurity phases [2,17,19].

The formation of well-crystallized NFPP in the composites NFPP/C and NFPP/rGO is further supported by the IR spectra (Figure 1d). In the region of 1250–400 cm^−1^, the IR spectra enable clear differentiation of the orthophosphate and diphosphate groups in the NASCON-type structure. The detailed assignment of the IR bands in NFPP was provided in our previous paper [15]. In this study, we compared the spectroscopic characteristics (i.e., band shape, intensities, and frequency positions) of NFPP and its carbon composites.

The comparison shows that NFPP/C and NFPP/rGO composites display the same vibrational characteristics as those of carbon-free NFPP (Figure 1d). It should be noted that the position deviation between 1 and 2 cm^−1^ falls in the limit of the experimental resolution. The similarity in the spectral features of the sample provides evidence of the identical local structure of PO_4_ and P_2_O_7_ groups in the three samples. Therefore, the formation of composites of NFPP with carbon black and rGO neither induces change in their anion frameworks nor oxidation processes related to the Fe^2+^ ions.

In addition, the IR spectra of NFPP/C and NFPP/rGO composites display bands that are associated with the nature of the carbon additives. The bands at 1573 cm^−1^ for NFPP/rGO and at 1630 cm^−1^ for NFPP/C (less intensive) are due to the in-plane stretching motion of pairs of *sp*^2^ carbon atoms (C=C) [25,26]. This vibration is typical for aromatic and olefinic molecules and always lies in the region of 1500–1630 cm^−1^ [26,27]. The position of this band in our NFPP/rGO is very close to that in ideal graphite (1588 cm^−1^, *E*_1*u*_ mode) [26,27] which is an indication of the more graphitic-like nature of the rGO material than that of carbon black [26,27,28]. The other IR active band in graphite appears at 868 cm^−1^ (*A*_2u_ mode), but in our spectra it is probably hidden by the strong absorptions of the P–O–P bridge at 884 cm^−1^ (Figure 1d). The band in the region 1460–1448 cm^−1^ (more intense in NFPP/C) could be attributed to the carbonate-related and C=N species [29,30].

Further information about the carbon structure can be obtained using Raman spectroscopy based on the intensity ratio of the D and G bands (I_D_/I_G_ ratio) that are characteristic for disordered carbonaceous materials [26,31]. The carbonaceous materials used (Super C65 carbon black and rGO (Graphit Kropfmühl GmbH)) are commercial products and they are well-characterized by Raman spectroscopy [32,33,34]. In our previous papers, we have presented the Raman spectrum of carbon black which exhibits two bands at 1590 and 1326 cm^−1^ assigned to G and D band, respectively [32,33]. The intensity ratio, I_D_/I_G_, measured from the areas of the bands is 1.75 (measured from the peaks intensity is 1.15) [32,33]. As reported by Ackermann et al., the Raman spectrum of rGO (Graphit Kropfmühl) also displays G and D bands at approximately 1590 and 1326 cm^−1^, the peak intensity ratio I_D_/I_G_ being 0.41 [34]. The comparison clearly shows that the level of disorder of carbon black is significantly larger than that of rGO. Moreover, we have established that the carbon structure in the composites between phosphates and Super C65 carbon black prepared via our ball-milling treatment remains unchanged [32,33]. Therefore, the conclusion derived from the Raman spectra of the pure carbon additives can be referred to the respective composites, i.e., the level of disorder of carbon black in NFPP/C composite is much higher than that of rGO in NFPP/rGO composite, in agreement with the IR spectra.

**Figure 2:**
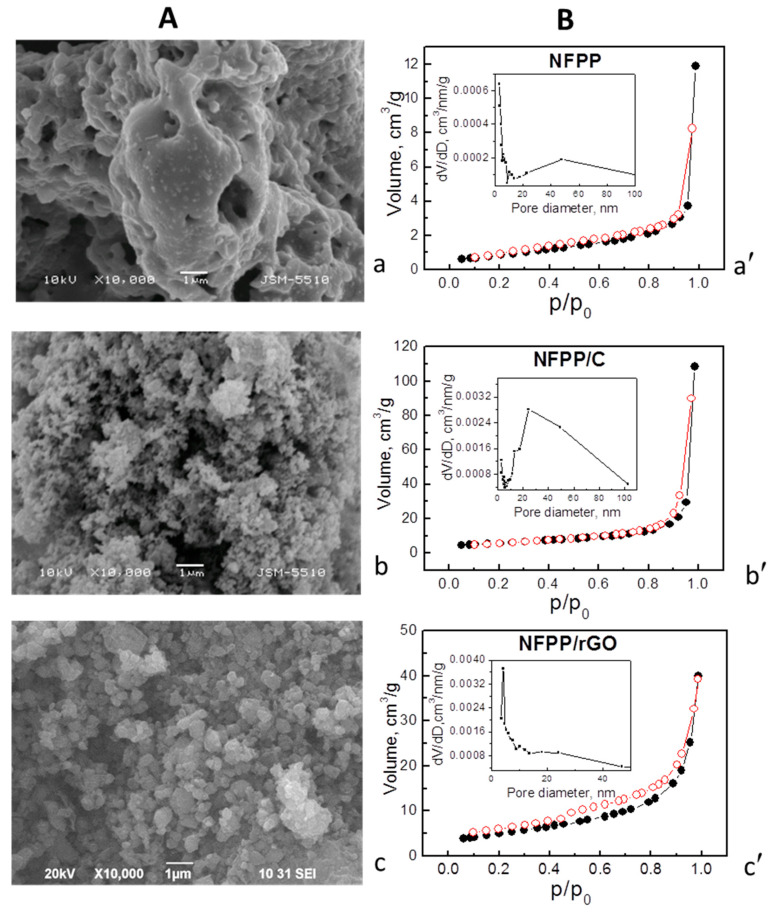
(**A**) SEM images and (**B**) nitrogen adsorption/desorption isotherms (full/open symbols) with pore size distributions (insets) of: NFPP (**a**,**a**′), NFPP/C (**b**,**b**′), and NFPP/rGO (**c**,**c**′).

Although the structural characteristics of NFPP are unaffected by the ball-milling with carbon additives, the morphology and porosity of NFPP/C and NFPP/rGO undergo some changes (Figure 2).

The morphology of pristine NFPP comprises irregular micrometric aggregates (above 10 μm), where individual particles are hardly visible (Figure 2a). The ball-milling with carbon additives effectively breaks these aggregates: for NFPP/C, nanoparticles prevail, while for NFPP/rGO, the smaller globe-like aggregates with sizes about 200 nm dominate (Figure 2b,c). It should be mentioned that the ball-milling of NFPP without carbonaceous materials is not energetically enough to break NFPP aggregates into small particles, as seen from Figure S1. The SEM image (Figure S1) demonstrates that, even after ball-milling, NFPP comprises mainly irregular aggregates with micrometer sizes (between 1 and 5 μm), but smaller particles or aggregates below 0.2 μm are also visible. The observed agglomeration could also be explained with a high surface tension of NFPP particles, but it can be effectively overcome by ball-milling with carbon additives (Figure 2A and Figure S1).

**Figure 3:**
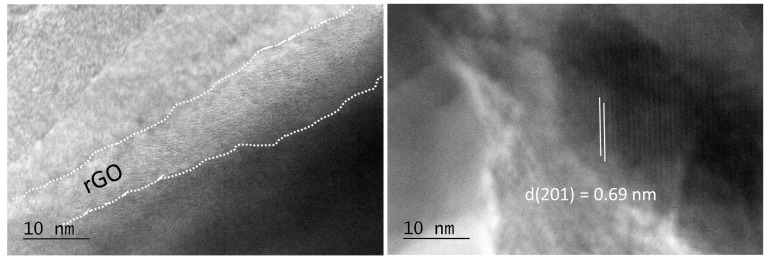
HR-TEM image of NFPP/rGO composite. The rGO layer is marked by white dotted lines.

The co-existence of NFPP and rGO in the composite was confirmed by HR-TEM (Figure 3). The thickness of the rGO shell is inhomogeneous and varies from about 10 to 30 nm. In addition, separate rGO particles are also visible. Inside the composites, the NFPP phase preserves its crystal structure intact (Figure 3), as was established from XRD and IR experiments. HR-TEM image exhibits lattice fringes from the (201) plane and the interplanar space of 0.69 nm coincides very well with that found via the XRD analysis (6.89 Å).

The ball-milling process is concomitant with variations in the textural characteristics together with preservation of the isotherm type: the isotherms are of type II with H3 hysteresis (presence of slit-shaped pores), which is typical of mesoporous materials [35,36,37]. As can be expected, the composites have considerably higher specific surface areas: 20 and 18 cm^2^/g for NFPP/C and NFPP/rGO, respectively, vs. 3 cm^2^/g for NFPP (Figure 2B), at least owing to the high specific surface areas of pure carbon additives (49 cm^2^/g for carbon black and 363 cm^2^/g for rGO, Figure S2). The total pore volumes increase as well: from 0.02 cm^3^/g in NFPP to 0.17 and 0.10 cm^3^/g in NFPP/C and NFPP/rGO, respectively. On the other hand, the pore volume for NFPP/rGO is rather low (even lower than that for NFPP/C), considering the much larger total pore volume of pure rGO (1.58 cm^3^/g, Figure S2). This result implies that during the ball-milling treatment a partial blocking of the slit-shaped mesopores of rGO by the NFPP particles (larger than 100 nm) occurs and information about this can be derived by the pore size distribution curves. For rGO, a homogeneous distribution of the mesopores in a narrow range of sizes between 3.5 and 6.5 nm is clearly visible (Figure S2); meanwhile, for composite NFPP/rGO the distribution appears to be bimodal: there is a predominant part of the pores between 3 and 10 nm and a less expressed part between 13 and 45 nm (Figure 2B). From these data, one could suppose that the small slit-shaped mesopores of rGO with sizes between 3.5 and 6.5 nm are most probably blocked by the NFPP particles to a high extent giving rise to a lowered pore volume and specific surface area of the composite NFPP/rGO. For comparison, carbon black is characterized by a broader pore size distribution with mesopores having diameters between 3 and 50 nm (Figure S2) and the pore blocking by the NFPP particles occurs to a lesser extent. The composite NFPP/C and pristine NFPP also exhibit a monomodal distribution of the pores within a broad range between 10 and 100 nm (Figure 2B).

### 3.2. Sodium Storage Properties of NFPP/C and NFPP/rGO Composites

**Figure 4:**
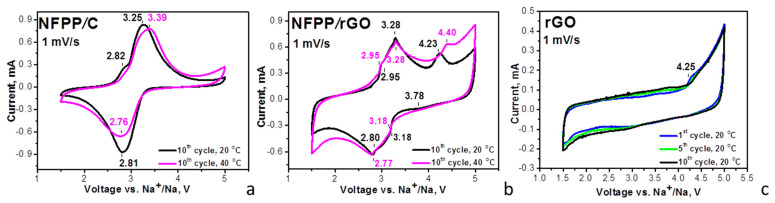
CV curves at a scanning rate of 1 mV/s for NFPP/C (**a**) and NFPP/rGO (**b**) in sodium-half cells. The cells operate firstly at 20 °C (10 cycles) and then at 40 °C (10 cycles). For the sake of comparison, the CV curves of rGO in sodium half-cells are also provided (**c**).

The capability of the composites to intercalate Na^+^ at elevated temperatures is compared in Figure 4.

In sodium-ion half cells (20 °C), the CV curves of the composites display broad oxidation and reduction peaks at around 3.25 and 2.80 V, respectively. These redox peaks coincide with those previously reported for NFPP and were attributed to the Na^+^ intercalation/deintercalation into the NASICON structure of NFPP owing to the Fe^2+^/Fe^3+^ ionic pair: Na_4_Fe_3_(PO_4_)_2_P_2_O_7_–3Na^+^ ↔ NaFe_3_(PO_4_)_2_P_2_O_7_ [2,4,10,15,17]. The carbon and rGO additives have no measurable effect on the redox peak due to the Na^+^ intercalation, which confirms the XRD data on the structure stability of NFPP after the ball-milling treatment (Figure 1). The close inspection of the CV profiles indicates that the redox peak due to Na^+^ intercalation is split into several components, the splitting being better expressed for the NFPP/rGO composite. It is worth mentioning that Na^+^ deintercalation is a step-wise process due to the availability of several crystallographic sites for sodium [2,4,17,22,24]. The sequence deintercalation of Na^+^ from one crystallographic site to another is resolved when sodium electrolytes containing NaTFSI [15] or NaClO_4_ [2,4,22,24] salts are used, while in NaPF_6_-based electrolyte, the Na^+^ deintercalation seems structureless [15]. It appears that, for the NFPP/rGO composite, the Na^+^ deintercalation remains step-wise even in the NaPF_6_-based electrolyte (Figure 4b). This can be related with the textural characteristics of NFPP/rGO, where specific bimodal pore-size distribution is observed (Figure 2B). These textural characteristics could be beneficial for better penetration of the electrolyte ions within the bulk of the electrodes, as well as being able to help with the formation of porous electrolyte–electrode interface, thus contributing to the step-wise intercalation of Na^+^.

By increasing the operating temperature from 20 to 40 °C, the oxidation and reduction peaks for the NFPP/C composite become broader and the peak-to-peak separation increases from 0.44 to 0.63 V (Figure 4a). This provides evidence that the elevated temperature provokes a suppression in the electron transfer reaction during the Na^+^ intercalation into the NFPP/C composite. In contrast to NFPP/C, the composite NFPP/rGO retains unchanged its CV curve profiles at 40 °C (Figure 4b). Again, this can be related to the textural characteristics: while the rGO additive facilitates the formation of a porous electrolyte–electrode interface, the carbon black additive has a negative effect.

Moreover, the CV curve of the NFPP/rGO composite brings some features of capacitive electrochemical storage in addition to the Faradaic ones, while the NFPP/C composite stores energy via the Faradaic reaction only (Figure 4). To understand this observation, Figure 4 also provides the CV curves of rGO used as a positive electrode in sodium half-cell (Figure 4c). The CV curve of rGO displays a rectangular-like profile, which is typical behavior of capacitance electrochemical storage. This is consistent with previous studies on the electrochemical behavior of rGO in sodium-ion devices, where the sodium storage was associated with diffusionless surface reactions [38,39]. Above 4.2 V, the enhancement in the oxidation curve implies the beginning of the interaction between rGO and electrolyte which requires further study. Comparing the CV profiles of the NFPP/rGO and rGO additive, it appears that the composite NFPP/rGO stores energy via a hybrid mechanism: the NFPP constituent contributes to the bulk Faradaic reaction, while the rGO additive initiates the capacitive surface reactions. This mechanism of bulk and surface reactions appears to be facilitated by the textural characteristics of NFPP/rGO composite.

**Figure 5:**
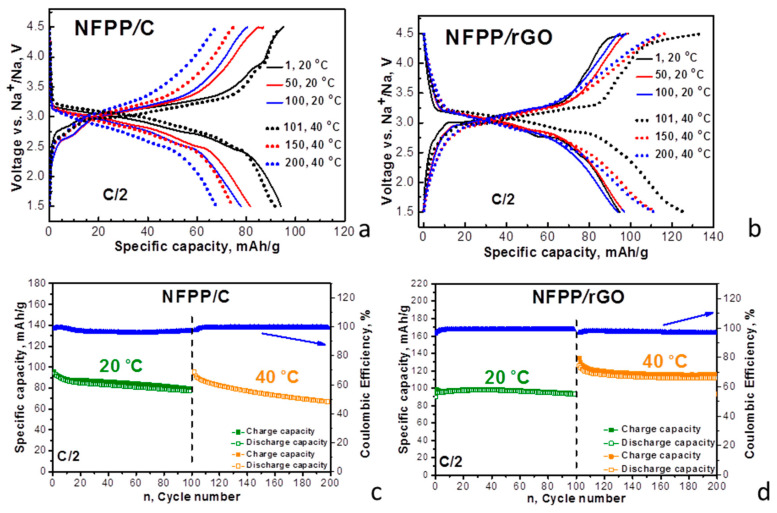
Charge/discharge curves of NFPP/C and NFPP/rGO in sodium half-cells (**a**,**b**). The cells operate firstly at 20 °C (cycles from 1 to 100) and then at 40 °C (cycles from 101 to 200). Cycling stability and Coulombic efficiency at C/2 rate of NFPP/C and NFPP/rGO in sodium half-cells at 20 and 40 °C (**c**,**d**).

Furthermore, the galvanostatic test of the composites supports the different electrochemical performance of NFFP/C and NFPP/rGO composites (Figure 5). The charge/discharge curves of NFFP/C and NFPP/rGO demonstrate a voltage plateau at around 3.0 V (Figure 5), as was observed in the CV curves (Figure 4). This plateau is related with Na^+^ intercalation to the NFPP structure. Irrespective of the similar charge/discharge curve profiles, the specific discharge capacity and the cycling stability at 20 °C of the composite with rGO exceed those of the composite with carbon black additive: 95 mAh/g vs. 80 mAh/g after the 100 cycles at a rate of C/2, the corresponding cycling stability being 95% vs. 84%. It is worth comparing our data on NFPP/rGO with that previously reported on NFPP decorated with rGO: at a rate of C/2, a reversible capacity of 117 mAh/g was reported [14]. We obtained a good storage performance of the composite NFPP/rGO using a simple and fast method of synthesis.

The difference in the electrochemical performance of NFPP/rGO and NFPP/C becomes more significant at 40 °C: a discharge capacity of 115 mAh/g vs. 70 mAh/g after the next 100 cycles at a rate of C/2, the corresponding cycling stability being 88% vs. 70% at 20 °C. It is of importance that the Coulombic efficiency varies between 97 and 100% for both NFPP/C and NFPP/rGO composites. The improved storage performance of the composite with rGO can be correlated with its hybrid mechanism of energy storage. In addition, as far as we know, we provide the first data on the sodium storage performance of NFPP/rGO at elevated temperatures.

### 3.3. Lithium Storage Properties of NFPP/C and NFPP/rGO Composites

In comparison with the Na-ion cell, the energy storage by NFPP in the Li-ion cell is a more complex process [2,15,17,18]. At OCV (i.e., before the electrochemical reaction), NFPP reacts partially with the Li-electrolyte including Na^+^/Li^+^ exchange reactions. Furthermore, the first charge/discharge cycle gives rise to extraction of the bigger Na^+^ and consequent insertion of the smaller Li^+^ ions. This reaction is concomitant with a change of Na-to-Li ratio in the electrolyte, but the total alkali content remains 1 M. After the next cycles, Li^+^ is preferentially intercalated leading to formation of the (Na_3−x_Li_x_)NaFe(PO_4_)_2_P_2_O_7_ phase [2,15,17,18].

**Figure 6:**
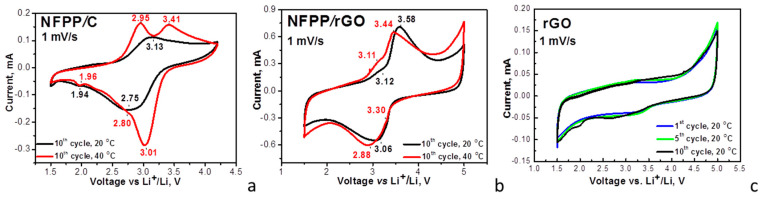
CV curves at a scanning rate of 1 mV/s for NFPP/C (**a**) and NFPP/rGO (**b**) in lithium half-cells. The cells operate firstly at 20 °C (10 cycles) and then at 40 °C (10 cycles). For the sake of comparison, the CV curves of rGO in lithium half-cells are also provided (**c**).

Lithium intercalation into composites was examined in lithium half-cells with a conventional carbonate-based electrolyte (i.e., 1 M LiPF_6_ in EC/DMC) (Figure 6).

For the NFFP/C composite, the CV curve at 20 °C displays a couple of broad oxidation/reduction peaks centered at around 3.13 V/2.75 V, which are split into two components at 40 °C: oxidation peaks at 2.95 V and 3.41 V and reduction peaks at 3.01 V and 2.80 V. For the NFPP/rGO composite, a broad oxidation peak centered at 3.58 V with a shoulder at 3.12 V together with a broad reduction peak at 3.06 V are resolved at 20 °C. The elevated operating temperature slightly changes the peak positions, but the curve profile is retained as a whole. Therefore, the operating temperature induces different electrochemical responses of NFPP/C and NFPP/rGO composites in both lithium and sodium ion cells. The textural properties are mainly responsible for the observed temperature-induced phenomenon.

The redox peaks observed for NFPP/C and NFPP/rGO composites coincide with those previously found for NFPP, which are due to the consecutive intercalation of Li^+^ into the NASICON structure [15,17]. This means that for the NFPP/C and NFPP/rGO composites, the Li^+^ intercalation into NFPP also takes place with the participation of the Fe^2+^/Fe^3+^ redox couple. As in the case of Na-ion cell, the appearance of a tail in CV curves above 4.2 V implies some side reactions between NFPP composites and the Li electrolyte.

The comparison of the CV curve profiles for NFPP/C and NFPP/rGO discloses the next peculiarity, which is associated with the mechanism of the lithium storage. Similar to sodium storage, the CV curve profile for NFPP/rGO evidences the simultaneous occurrence of capacitive and Faradaic electrochemical reactions, while only Faradaic reactions take place for the NFPP/C composite. This is supported by the observed rectangular-like profile of the CV curve of rGO in lithium-half cells that is characteristic of capacitive reactions (Figure 6c). Therefore, the CV data reveal that the NFPP/rGO composite stores lithium via a hybrid electrochemical mechanism, as we established for sodium storage.

**Figure 7:**
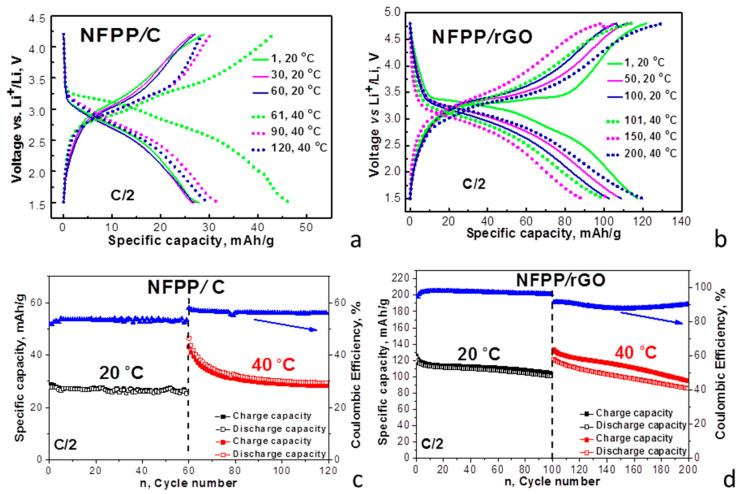
Charge/discharge curves of NFPP/C and NFPP/rGO in lithium half-cells (**a**,**b**). The cells operate firstly at 20 °C, and then at 40 °C. Cycling stability and Coulombic efficiency at C/2 rate of NFPP/C and NFPP/rGO in lithium half-cells at 20 and 40 °C (**c**,**d**).

The different mechanism of the lithium storage determines the charge/discharge characteristics of the composites (Figure 7).

At room temperature, the charge/discharge curves of the composites consist of a voltage plateau at 3.2 V vs. Li/Li^+^. However, NFFP/C and NFPP/rGO composites deliver completely different specific capacity: 28 mAh/g for NFFP/C and 123 mAh/g for NFPP/rGO. These data are also confirmed by the specific capacity determined from the CV curves: 31 mAh/g for NFFP/C and 107 mAh/g for NFPP/rGO. Furthermore, the cycling stability is also different: about 93% for NFPP/C and 89% for NFPP/rGO after 60 cycles (85% for NFPP/rGO after 100 cycles). However, the Coulombic efficiency tends to 99% for both NFPP/C and NFPP/rGO. We think that the excellent performance of NFPP/rGO as compared to NFPP/C is a result of the versatile role of the rGO additive: (i) rGO ensures a high electrical conductivity; (ii) rGO seems to wrap well NFPP particles thus changing the textural parameters (Figure 2 and Figure 3); and (iii) rGO displays capacitive reactions (Figure 6c).

At an elevated temperature (i.e., 40 °C), the shape of the charge/discharge curves remains constant, but the performance becomes worse for both composites. For NFPP/C, the specific capacity increases at 40 °C from 28 to 48 mAh/g at the expense of worsened cycling stability (i.e., a decrease from 93% to 83%). For NFPP/rGO, the specific discharge capacity at 40 °C is close to that determined at 20 °C (121 mAh/g), but the cycling stability decreases reaching a value of about 71%. An important sign for worsened electrochemical performance of the composites at elevated temperature is the Coulombic efficiency: for NFPP/C, the Coulombic efficiency exceeds 100%, while for NFPP/rGO, the Coulombic efficiency varies around 90%. This provides evidence of the occurrence of side reactions when NFPP/C and NFPP/rGO composites work at elevated temperatures in lithium-ion cells.

### 3.4. Ex Situ Studies

**Figure 8:**
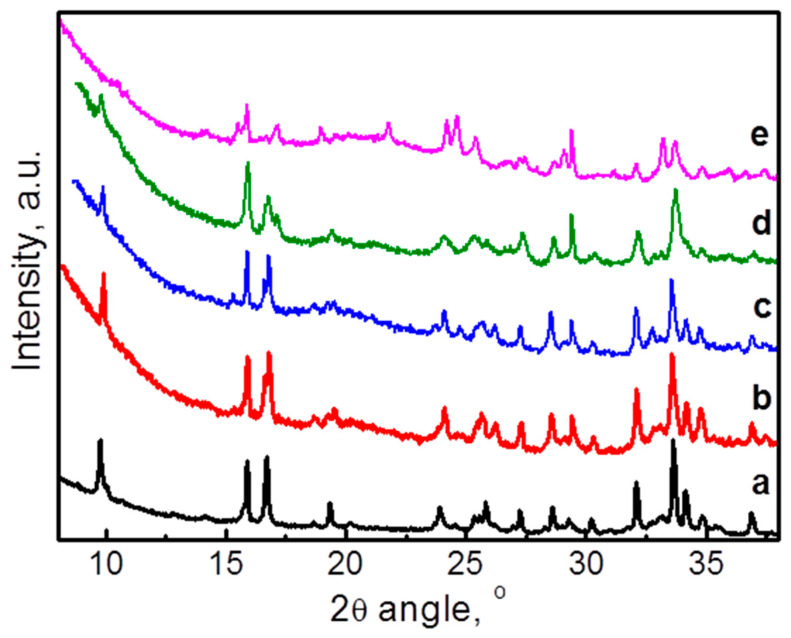
Ex situ XRD patterns of NFPP/C (**a**) and electrodes NFPP/C and NFPP/rGO cycled in Na half-cells (**b**,**c**), NFPP/C and NFPP/rGO cycled in Li half-cells (**d**,**e**) for 200 cycles (100 cycles at 20 °C and subsequent 100 cycles at 40 °C).

The structural changes during the prolonged electrochemical oxidation/reduction reactions in NFPP/C and NFPP/rGO electrodes in Na- and Li half-cells are followed by ex situ XRD. The electrochemical cells were switched off at 1.5 V (discharged state) after 200 cycles (100 cycles at 20 °C and subsequent 100 cycles at 40 °C) and the electrodes were analyzed. Since the XRD patterns of pristine NFPP/C and NFPP/rGO composites are practically the same, in Figure 8, only the XRD pattern of NFPP/C is given for comparison with these of the cycled electrodes. It can be seen that after 200 cycles there is no substantial change in the XRD patterns of both NFPP/C and NFPP/rGO compared with the pristine state, particularly in the Na half-cells. The diffraction peaks are indexed in the same orthorhombic crystal lattice as pristine NFPP with approximately the same lattice parameters (Table 1). This feature indicates a high structural stability of both NFPP/C and NFPP/rGO electrodes even at prolonged cycling at elevated temperature of 40 °C. The structural stability is an important advantage of NFPP electrode. It has been demonstrated that the Na^+^ extraction/insertion into NFPP proceeds topotactically within the framework of one-phase with low volume change (i.e., of about 4%) [2,4,11,17,24].

Contrary to the Na half-cells, in Li half-cells the XRD patterns exhibit some changes in the peak positions and intensities (Figure 8c,d), which is related to the intercalation of smaller Li^+^ instead of bigger Na^+^. However, the quality of the XRD patterns is not good enough for their indexing. On the other hand, the low quality of the XRD patterns could serve as an indirect sign for the amorphization of NFPP-based composites when they work in Li half-cells, which in turn determines the worse performance of the composites in Li half-cells than those in Na half-cells. This is especially valid for the NFPP/rGO composite.

**Figure 9:**
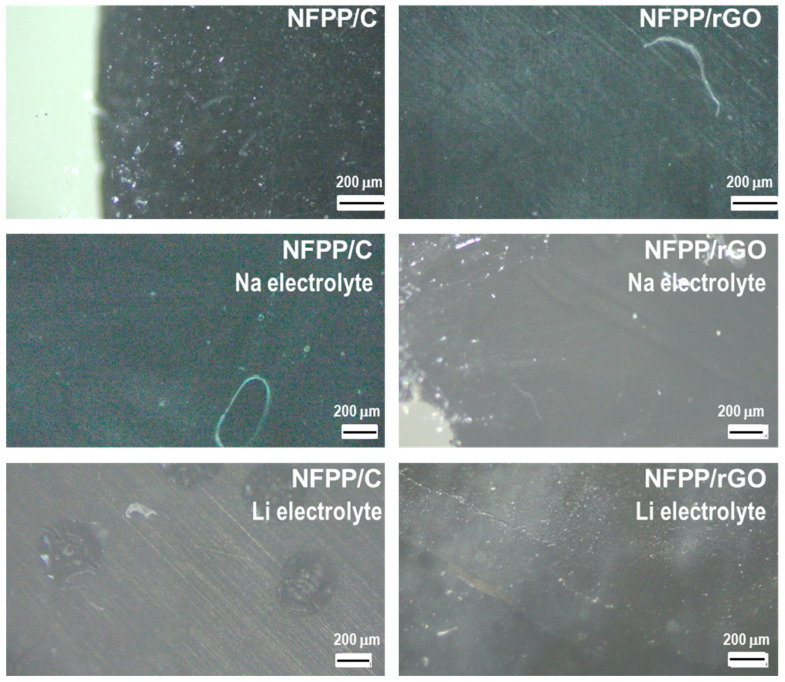
Optical images of initial NFPP/C and NFPP/rGO electrodes and after cycling in Na half-cells and Li half-cells for 200 cycles (100 cycles at 20 °C and subsequent 100 cycles at 40 °C).

**Figure 10:**
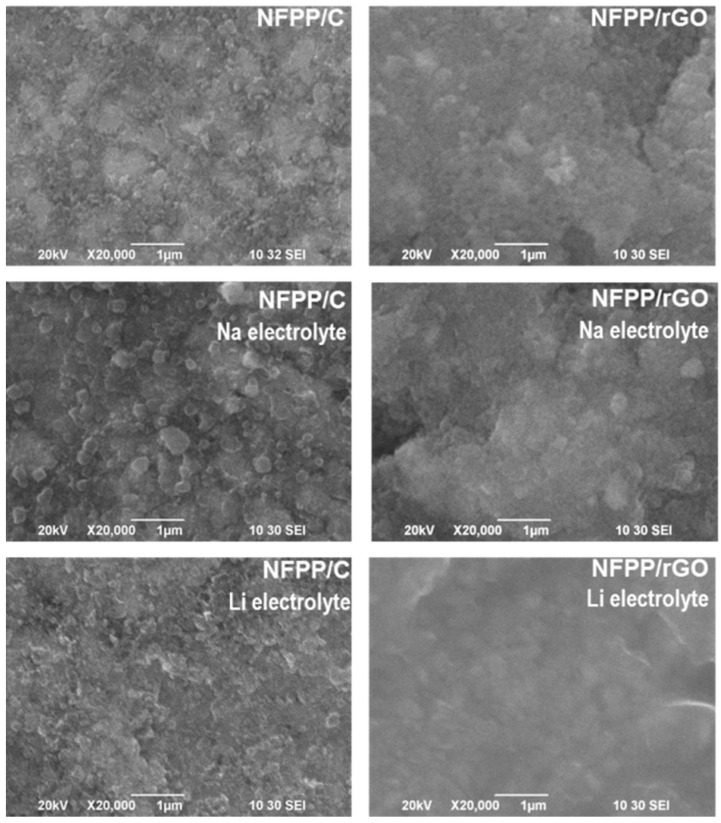
SEM images of initial NFPP/C and NFPP/rGO electrodes and after cycling in Na half-cells and Li half-cells for 200 cycles (100 cycles at 20 °C and subsequent 100 cycles at 40 °C).

Furthermore, the surface of the same cycled electrodes was observed using optical microscopy and SEM (Figure 9 and Figure 10). In general, the electrode roughness seems unchanged after the electrode cycling in both Na and Li half-cells (Figure 9). The preservation of the electrode roughness sheds further light on the process of loss of crystallinity of the composites after working in Li half-cells: it appears that the amorphization of the electrode proceeds without any dissolution reactions. At higher magnification, a veil on electrode composites working in Li half-cells becomes visible, especially for NFPP/rGO (Figure 10). This could be tentatively related to the formation of a specific cathode–electrolyte interface, which deserves further studies. In comparison with Li half-cells, the electrodes working in Na half-cells seem to retain their morphology characteristics unchanged (Figure 9 and Figure 10).

## 4. Conclusions

Ball milling followed by thermal treatment in Ar atmosphere at 400 °C yields composites of NFPP with carbon black and rGO. In these composites, NFPP retains its NASICON-type structure unchanged. The carbon additives affect the morphology and texture of the composites. The carbon black enables the exposure of nanoparticles of NFPP, while rGO causes the formation of globe-like aggregates with sizes of about 200 nm. The NFPP/C composite exhibits a monomodal pore size distribution between 10 and 100 nm, while a bimodal pore size distribution in the ranges of 3–10 nm and 13–45 nm occurs for NFPP/rGO.

In sodium half-cells, the composite of NFPP with rGO outperforms that with carbon black: at 20 °C, the reversible capacity is 95 mAh/g with a cycling stability of 95% after 100 cycles at a rate of C/2; meanwhile, at 40 °C, the reversible capacity increases to about 115 mAh/g at the expense of the lowered cycling stability to 88%. The improved performance of the NFPP/rGO composite is a result of the hybrid mechanism of Na-storage: although NFPP ensures the Na-storage via bulk Faradaic reactions, rGO gives rise to capacitive surface reactions. The hybrid mechanism of Na storage could also be correlated with the specific textural characteristics of NFPP/rGO composite.

In lithium half-cells, the composite NFPP/rGO stores Li via a hybrid mechanism, while NFPP/C operates via standard Li^+^ intercalation reactions. As a result, a higher reversible capacity is achieved for the composite with rGO, but the cycling stability of the composite with carbon black is better. However, at elevated temperatures, the performance of both NFPP/C and NFPP/rGO becomes worse due to the initiation of side reactions with the electrolytes. Further studies are needed to suppress these reactions, which will be of importance for improving the performance of the composites in Li ion cells at elevated temperatures.

In general, this study reports the first data on Na storage performance of composites of NFPP with rGO at elevated temperatures, thus revealing the path for their commercialization.

## Figures and Tables

**Table 1:** Lattice parameters of powder NFPP, carbon composites NFPP/C and NFPP/rGO (SG *Pn*2_1_*a*), and electrodes cycled in NaPF_6_/PC electrolyte and stopped at 1.5 V. For comparison, the literature data for NFPP/C and NFPP/rGO obtained using different methods are also provided.

Description	*a,* Å	*b,* Å	*c*, Å	*V,* Å^3^
NFPP	18.1018(6)	6.5331(2)	10.6452(3)	1258.93(7)
NFPP/C	18.0863(8)	6.5390(3)	10.6461(4)	1259.09(10)
NFPP/rGO	18.0854(6)	6.5345(6)	10.6467(7)	1259.95(12)
*Electrodes cycled in NaPF_6_/PC electrolyte, analyzed in discharged state (1.5 V)*				
NFPP/C (200 cycles)	18.0711	6.5340	10.6626	1259.02
NFPP/rGO (200 cycles)	18.0838	6.5344	10.6428	1257.63
*Literature data for NFPP/C and NFPP/rGO obtained by different methods*				
NFPP/C [10]: template method	18.0604(6)	6.5354(3)	10.6533(7)	1257.446(6)
NFPP/C [24]: sol-gel	18.1953(5)	6.5639(3)	10.6965(4)	1277.50
NFPP/C [16]: spray-drying	17.938	6.507	10.623	1239.94
NFPP/C [6]: solution combustion	18.038(4)	6.5429(13)	10.6744(17)	1259.8(5)
NFPP/rGO [14]: spray-drying	17.9675	6.5402	10.6672	1253.51

## Data Availability

Data available on request.

## Supplementary Materials

The following supporting information can be downloaded at: https://www.mdpi.com/article/10.3390/ma16196546/s1, Figure S1: SEM image of ball-milled NFPP without carbonaceous additives; Figure S2: Nitrogen adsorption/desorption isotherms (full/open symbols) with pore size distributions (insets) of carbon black and rGO.
