# Supplementary material for: Carbon-Based Composites with Mixed Phosphate-Pyrophosphates with Improved Electrochemical Performance at Elevated Temperature
Source: Materials (Basel). 2023 Oct 4;16(19):6546. doi: 10.3390/ma16196546 (PMC10574593; doi:10.3390/ma16196546)
Supplement: Supplementary file 1 [file materials-16-06546-s001.zip › materials-2632904-supplementary.pdf]

Carbon-Based Composites with Mixed Phophate-Pyrophosphates with Improved Electrochemical Performance at Elevated Temperature

Sonya Harizanova, Trajche Tushev, Violeta Koleva* and Radostina Stoyanova*

Institute of General and Inorganic Chemistry, Bulgarian Academy of Sciences, 1113 Sofia, Bulgaria
*Correspondence: vkoleva@svr.igic.bas.bg (V.K.), radstoy@svr.igic.bas.bg (R.S.);
Tel.: +359-979-3915

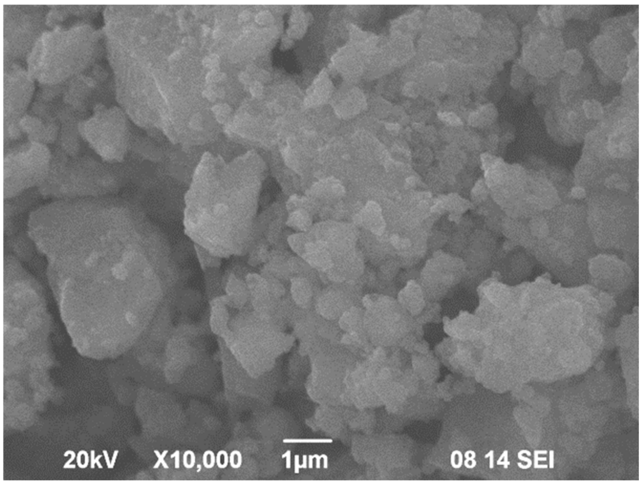

Figure S1. . SEM image of ball-milled NFPP without carbonaceous additives

The SEM image (Fig. S1) demonstrates that, even after ball-milling, NFPP comprises mainly irregular aggregates with micrometer sizes (between 1 and 5 μm), but smaller particles or aggregates below 0.2 μm are also visible. This means that the ball-milling is not energetically enough to broken NFPP aggregates into small particles, but the observed NFPP agglomeration can be effectively overcome using carbon additives.

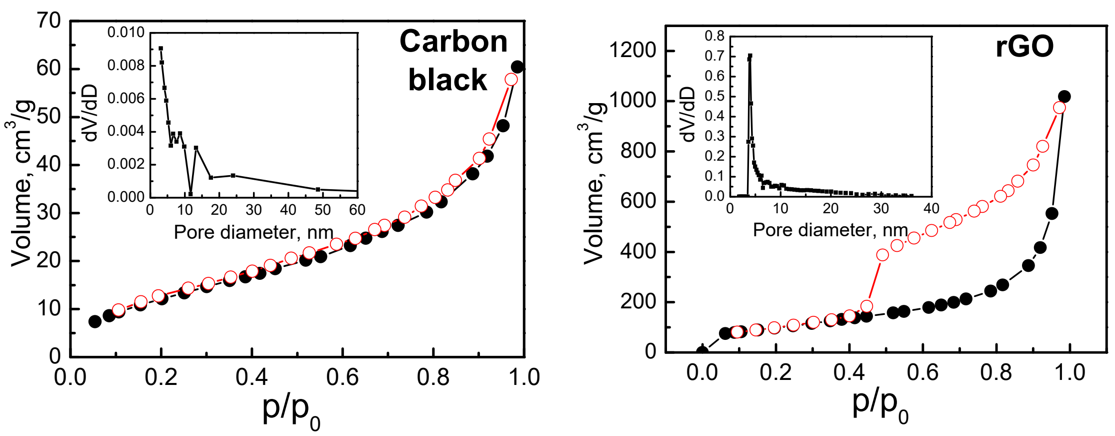

Figure S2. Nitrogen adsorption/desorption isotherms (full/open symbols) with pore size distributions (insets) of carbon black and rGO.

Both carbon black and rGO are typical mesoporous materials (the isotherms being of type II with H3 hysteresis), but with significantly different porous textures. The specific surface area is 49 cm^2/g for carbon black and 363 cm^2/g for rGO. Accordingly, the total pore volume of rGO is considerably larger than that of carbon black: 1.58 cm^3/g vs. 0.09 cm^3/g. Moreover, rGO is characterized by a narrow pore size distribution with main part of mesopores having diameters between 3.5 and 6.5 nm, while a browder pore size distribution is available for carbon black with pores distributed between 3 and 50 nm.
